# Recent advances in immunotherapy for bladder cancer: mechanisms, clinical applications, and future perspectives

**DOI:** 10.3389/fonc.2026.1786965

**Published:** 2026-03-16

**Authors:** Rui Liu, Junlong Wang

**Affiliations:** 1 Department of Emergency Medicine, The Second Affiliated Hospital of Zhejiang Chinese Medical University, Hangzhou, China; 2 Department of Urology, The First Affiliated Hospital of Zhejiang Chinese Medical University (Zhejiang Provincial Hospital of Chinese Medicine), Hangzhou, China

**Keywords:** antibody–drug conjugates, biomarkers, bladder cancer, CAR-T cell therapy, immune checkpoint inhibitors, immunotherapy, tumor microenvironment

## Abstract

The past decade has witnessed a paradigm shift in the treatment of bladder cancer, propelled by significant advances in immunotherapy. Immune checkpoint inhibitors (ICIs) targeting PD-1/PD-L1 and CTLA-4, adoptive cellular therapies including chimeric antigen receptor T-cell (CAR-T) therapy, oncolytic viruses, and novel immunomodulatory agents have transformed the therapeutic landscape for both non–muscle-invasive bladder cancer (NMIBC) and advanced urothelial carcinoma (UC). This review provides a comprehensive analysis of recent advances in bladder cancer immunotherapy, with a focus on underlying molecular and cellular mechanisms, key clinical trial evidence, and emerging resistance pathways. We highlight the rapidly expanding therapeutic roles of ICIs, alongside innovative modalities such as CAR-T cell therapy directed against tumor-associated antigens—including NECTIN4, PSMA, and FRα. Emerging immunotherapeutic targets and therapeutic modalities are comprehensively reviewed. We critically evaluate key clinical trials and systematically assess combination strategies—including ICIs combined with chemotherapy, radiotherapy, targeted therapy, or antibody–drug conjugates (ADCs). Key determinants of the tumor microenvironment (TME)—such as immunosuppressive cell populations, regulatory cytokines, and metabolic barriers—are examined in the context of their roles in mediating therapeutic resistance. Biomarkers predictive of treatment response—including PD-L1 expression and tumor mutational burden—are summarized, integrating recent clinical and translational evidence. We conclude by outlining future research directions focused on overcoming therapeutic resistance, refining predictive and prognostic biomarkers, and developing next-generation immunotherapies to improve clinical outcomes for patients.

## 1 Introduction

Bladder cancer is a highly prevalent malignancy; in the United States, an estimated 84,870 new cases are projected to be diagnosed in 2025, with approximately 90% classified as urothelial carcinoma (UC). Among these cases, roughly 70% are diagnosed as non–muscle-invasive bladder cancer (NMIBC) (1). Historically, intravesical Bacillus Calmette-Guérin (BCG) immunotherapy has served as the cornerstone treatment for high-risk NMIBC, markedly reducing both recurrence rates and the risk of disease progression. However, a substantial proportion of NMIBC cases recur or progress despite BCG therapy; in particular, patients with BCG-unresponsive carcinoma *in situ* (CIS) face a significantly elevated risk of progression to muscle-invasive disease. Although radical cystectomy remains the standard curative intervention for refractory NMIBC, it is associated with considerable morbidity and long-term functional and quality-of-life implications (1).

In advanced UC, platinum-based chemotherapy has long served as the standard first-line treatment; however, median overall survival remains limited, at approximately 12–15 months (2). The advent of immune checkpoint inhibitors (ICI) has substantially transformed the therapeutic landscape. In 2017, the U.S. Food and Drug Administration (FDA) approved several PD-1/PD-L1 inhibitors—including pembrolizumab, nivolumab, atezolizumab, durvalumab, and avelumab—for the treatment of metastatic UC, based on clinical trial data demonstrating durable responses in select patient subgroups (2, 3). Maintenance with avelumab following chemotherapy (JAVELIN Bladder 100 trial) has established a new standard of care, significantly extending overall survival (OS) (4). More recently, the combination of enfortumab vedotin (EV)—an antibody–drug conjugate (ADC) targeting NECTIN4—and pembrolizumab has been approved for first-line treatment of cisplatin-ineligible patients with metastatic UC (5, 6).

In this review, we comprehensively delineate the mechanisms underlying immunotherapies for bladder cancer, critically appraise recent clinical trial evidence, and delineate emerging research frontiers. We systematically examine key therapeutic modalities—including ICIs (targeting PD-1, PD-L1, CTLA-4, and emerging novel checkpoints), cellular therapies (with emphasis on chimeric antigen receptor T-cell (CAR-T) approaches), oncolytic viruses, and intravesical immunotherapeutic strategies—as well as resistance factors in the tumor microenvironment, predictive biomarkers, and combination regimens. Particular attention is given to advances reported between 2022 and 2025, supported by citations from high-impact oncology and immunology journals, clinical trial reports, and official regulatory agency summaries.

## 2 Mechanisms of immunotherapy in bladder cancer

**Figure 1:**
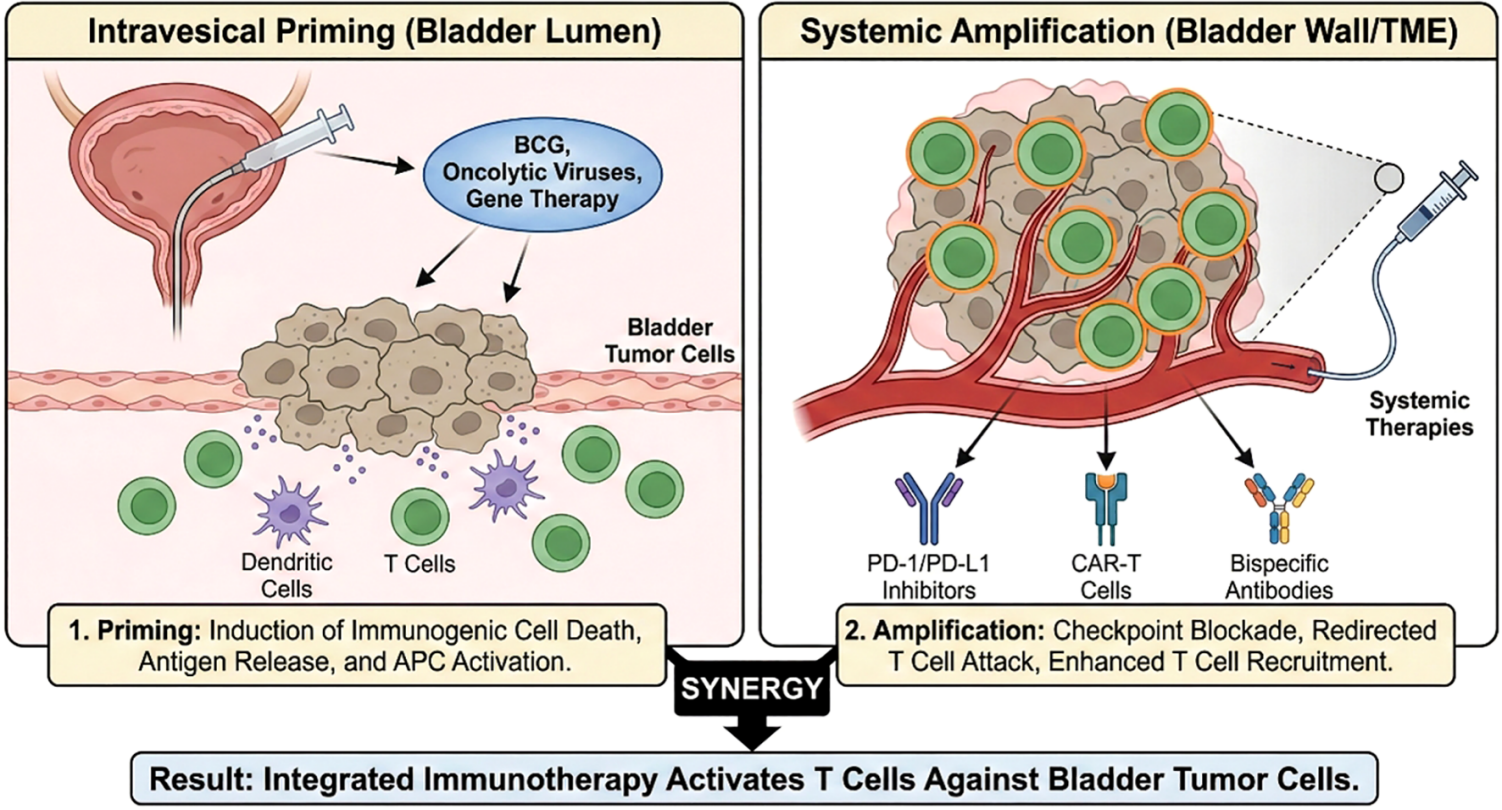
Integrated intravesical and systemic immunotherapy strategies for bladder cancer. Intravesical agents-including BCG and oncolytic viruses-prime antigen-specific T cells within the bladder microenvironment, and these primed T cells are subsequently expanded or reactivated by systemic immune checkpoint inhibitors or CAR-T cells, yielding synergistic antitumor responses.

Bladder cancers are frequently highly immunogenic—a feature largely attributable to their elevated tumor mutational burden (TMB) and prior exposure to carcinogens, such as tobacco smoke and industrial chemicals, which promote the generation of tumor-specific neoantigens. The UC tumor microenvironment (TME) constitutes a complex cellular ecosystem composed of tumor cells, stromal elements—including fibroblasts and vasculature—and various immune cells (T lymphocytes, macrophages, dendritic cells, myeloid-derived suppressor cells (MDSCs), and regulatory T cells (Tregs)). Effective immunotherapy must counteract the immunosuppressive characteristics of the TME. Key mechanisms include:

- Immune checkpoint pathways: Tumor cells upregulate PD-L1 and PD-L2 to engage PD-1 on T cells, thereby suppressing T-cell activation. CTLA-4 on Tregs competes with CD28 for binding to CD80 and CD86 on dendritic cells, thereby inhibiting naive T-cell priming (2, 3). Therapeutic blockade of these immune checkpoints using monoclonal antibodies—such as anti-PD-1, anti-PD-L1, and anti-CTLA-4 agents—reverses T-cell suppression and restores antitumor cytotoxic activity (2, 3). Clinical benefit has been demonstrated with agents including nivolumab, pembrolizumab, atezolizumab, durvalumab, avelumab.
- Effector T cells and cytokines: The presence of intratumoral CD8^+^ T cells is associated with favorable therapeutic response; conversely, immunosuppressive cytokines—such as TGF-β and IL-10—secreted by stromal or regulatory immune cells can potently suppress antitumor immunity (7, 8). For instance, heightened TGF-β signaling in cancer-associated fibroblasts has been correlated with poor response to ICIs (7). Therapeutic strategies that modulate the local cytokine milieu—including intravesical delivery of IFN-α via gene therapy (9) and administration of IL-15 agonists (1) —can enhance antitumor immune activation.
- Antigen presentation and neoantigens: A high neoantigen burden—often attributable to smoking-associated mutagenesis—enhances the immunogenic visibility of bladder tumors to cytotoxic T cells. However, genomic alterations—such as loss of antigen-presenting machinery and JAK1/2 mutations—can confer resistance to T cell–mediated immunity (3). Vaccines or adoptive T-cell therapies aim to broaden antigen-specific responses (discussed below).
- Intravesical priming: Local therapies—including BCG and oncolytic viruses—not only directly eliminate tumor cells but also potently activate innate immunity (e.g., via TLR signaling and dendritic cell maturation), thereby inducing localized inflammation of the bladder wall and facilitating robust T-cell infiltration (10, 11). This intravesical priming strategy can synergize effectively with systemic immunotherapies, as illustrated in **Figure 1**.

## 3 Immunotherapy in bladder cancer

### 3.1 Immune checkpoint inhibitors

#### 3.1.1 PD-1/PD-L1 inhibitors

**Figure 2:**
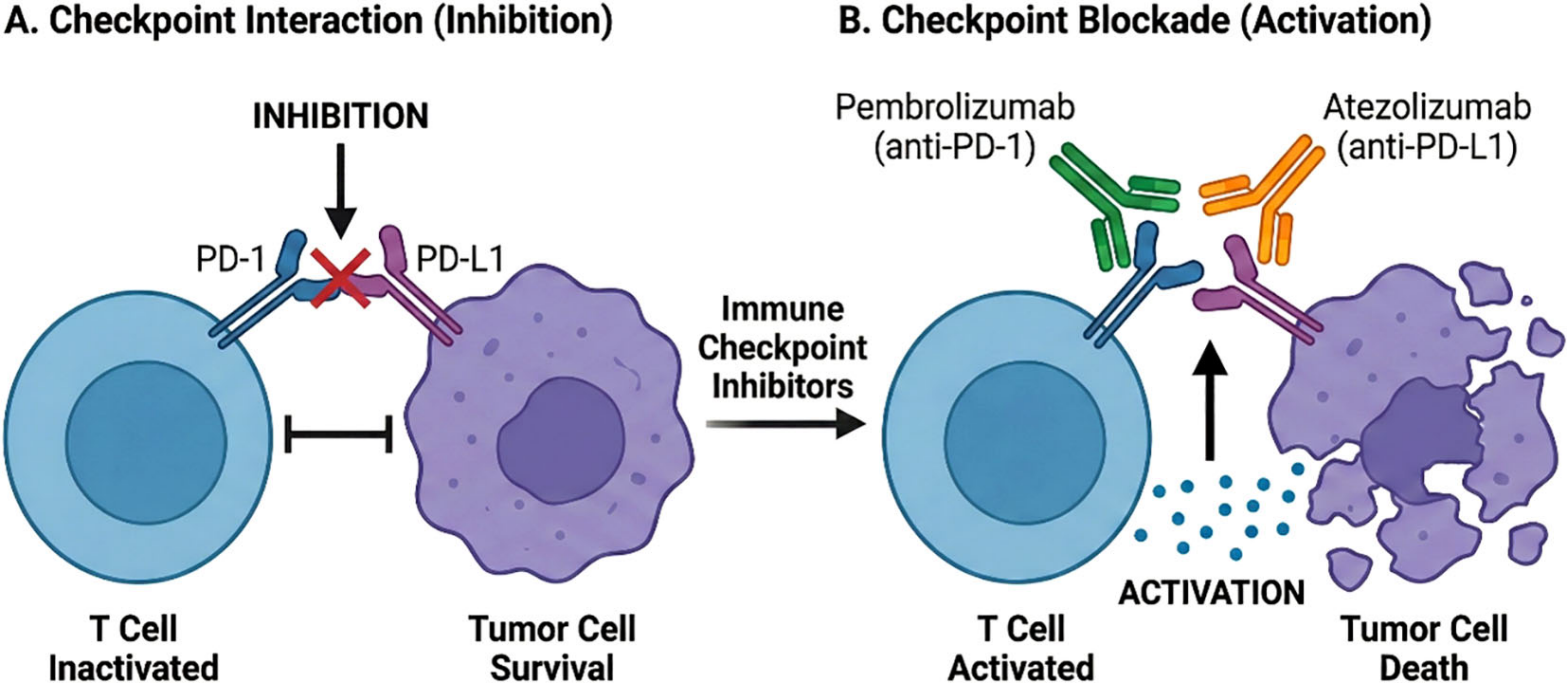
Schematic representation of immune checkpoint inhibitor in bladder cancer. **(A)** PD-1/PD-L1 interaction inhibits T-cell activity, promoting tumor cell survival. **(B)** Monoclonal antibodies (e.g., pembrolizumab, atezolizumab) block this interaction, leading to T-cell activation and tumor cell killing.

The most well-established immunotherapies for bladder cancer are monoclonal antibodies targeting PD-1 or PD-L1 (**Figure 2**). To date, five such agents have received approval from the U.S. FDA: pembrolizumab and nivolumab (anti-PD-1 antibodies), and atezolizumab, durvalumab, and avelumab (anti-PD-L1 antibodies). Their approved indications encompass a range of clinical settings:

- Second-line/metastatic after platinum-based chemotherapy: Pembrolizumab was the first ICI approved for metastatic UC after platinum-based therapy, based on the KEYNOTE-045 trial, which demonstrated superior overall survival (OS) compared with chemotherapy. Atezolizumab and nivolumab have also shown clinical benefit in platinum-refractory UC, however, the confirmatory phase III (IMvigor211 trial) yielded mixed results (2).
- First-line metastatic: In cisplatin-ineligible patients, pembrolizumab or atezolizumab monotherapy is approved for those with high tumor PD-L1 expression. However, trials of ICI versus chemotherapy in the general first-line setting (KEYNOTE-361 trial, IMvigor130 trial) were negative (3). Accordingly, current guidelines favor platinum-based chemotherapy followed by maintenance avelumab (JAVELIN Bladder 100 trial) for fit patients (4).
- Maintenance therapy: The phase III JAVELIN Bladder 100 trial established avelumab as a maintenance treatment following first-line chemotherapy, significantly improved median OS, extending it from 14.3 to 21.4 months (HR 0.69, *P* < 0.001) (4). Based on these findings, avelumab maintenance therapy is now considered the standard of care for eligible patients.
- Adjuvant/neoadjuvant therapy: In the CheckMate-274 trial, adjuvant nivolumab significantly improved disease-free survival (DFS) compared with placebo following radical cystectomy in patients with high-risk urothelial carcinoma (HR 0.70) (12, 13), leading to U.S. FDA approval for adjuvant use in this population. Early results from the AMBASSADOR trial evaluating adjuvant pembrolizumab are promising (14). Neoadjuvant use of ICIs (with or without chemotherapy) remains under active study, but so far KEYNOTE-866 trial (pembrolizumab + chemotherapy) failed to show clinical benefit (3).
- Intravesical and NMIBC: For patients with BCG-unresponsive CIS of the bladder (non–muscle invasive), pembrolizumab is FDA-approved based on results from the KEYNOTE-057 trial, which yielded a 41% complete response rate at 3 months (8). Other intravesical approaches include gene therapy (nadofaragene firadenovec) and the IL-15 agonist N-803 as adjuvants to BCG, as discussed below.

Clinical trial highlights: In the phase III IMvigor130 trial, atezolizumab added to chemotherapy conferred a modest improvement in progression-free survival (PFS) but did not significantly improve OS (15). Similarly, the addition of pembrolizumab to chemotherapy in the KEYNOTE-361 trial failed to yield statistically significant improvements in clinical outcomes (16). However, emerging regimens are reshaping clinical practice: the EV-302 trial (KEYNOTE-A39) recently demonstrated that first-line enfortumab vedotin (EV) plus pembrolizumab in cisplatin-ineligible patients led to a substantial improvement in OS (median 31.5 vs. 16.1 months, HR 0.47) (5, 6). These compelling results supported FDA approval of the EV–pembrolizumab combination in 2023. Consistent with these findings, the phase Ib EV-103 study reported an objective response rate (ORR) of 67.8% with the EV–pembrolizumab combination—markedly higher than the ORRs observed with EV monotherapy (45.2%) or pembrolizumab monotherapy (28.9%) in comparable patient populations (6).

PD-L1 as a biomarker: Tumor PD-L1 expression remains the most extensively investigated predictive biomarker for ICIs, however, its clinical utility is inherently limited. Different trials used varying assays and cutoffs, and PD-L1 expression can be heterogeneous and dynamic. Meta-analyses show that PD-L1-high tumors have higher response rates, yet a clinically meaningful proportion of PD-L1–negative patients still derive benefit from ICI therapy (2, 8). While regulatory agencies frequently require PD-L1 testing to guide first-line treatment decisions in cisplatin-ineligible settings, in practice, PD-L1 status serves only as an imperfect and context-dependent prognostic and predictive tool (2).

#### 3.1.2 CTLA-4 and novel checkpoints

CTLA-4 blockade (e.g., ipilimumab, tremelimumab) has not demonstrated clinically meaningful efficacy as monotherapy in UC, however, combination strategies are actively under study. The DANUBE trial—evaluating first-line durvalumab alone or in combination with tremelimumab—did not meet its primary endpoint of OS improvement. Nevertheless, a subset analysis revealed a potential benefit in patients with PD-L1–positive tumors (3). The CheckMate-901 and CheckMate-908 trials—assessing the combination of ipilimumab and nivolumab—remain ongoing or have recently reported preliminary findings: an interim press release indicated clinical activity for nivolumab plus chemotherapy, whereas the ipilimumab–nivolumab combination failed to meet its co-primary endpoints in the PD-L1–high population (3). Other emerging immune checkpoint targets—including LAG-3, TIM-3, and TIGIT—are being evaluated in clinical trials. For instance, the RELATIVITY-047 trial demonstrated the efficacy of relatlimab (an anti–LAG-3 antibody) combined with nivolumab in melanoma; analogous combination approaches are now being explored in UC. Additionally, next-generation ICIs targeting alternative immunomodulatory pathways—such as KIR and VISTA—are in early-stage development (8).

### 3.2 Adoptive cellular therapy

#### 3.2.1 Chimeric antigen receptor T-cell therapy

CAR-T cells are genetically modified T lymphocytes engineered to recognize tumor-associated antigens. Although CAR-T therapy has transformed the treatment landscape for hematologic malignancies, its application in solid tumors—including bladder cancer—remains in its early stages. Recent studies are actively exploring CAR-T therapies targeting UC-specific antigens.

- NECTIN-4–targeted CAR-T therapy: NECTIN-4 is a cell adhesion molecule that is highly expressed in UC—the same target of enfortumab vedotin. A recent study reported the development of a NECTIN-4-directed CAR-T cell therapy exhibiting potent antitumor activity. Notably, the study further demonstrated that pharmacologic activation of peroxisome proliferator-activated receptor gamma (PPARγ) upregulates NECTIN-4 expression, and that combination with the PPARγ agonist rosiglitazone significantly enhanced the cytotoxic efficacy of NECTIN-4–targeted CAR-T cells against bladder cancer cells (17). These findings support a novel combinatorial strategy to augment CAR-T cell potency in UC.
- HER2-targeted CAR-T therapy: Although HER2 expression in UC is heterogeneous, it is clinically targeted by the ADC disitamab vedotin (RC48). Preclinical studies demonstrate that HER2-specific CAR-T cells exhibit potent cytotoxic activity against UC cells; however, clinical efficacy data are still pending.
- NKG2D CAR-T therapy: A first-in-human clinical trial (CYAD-01) targeting NKG2D ligands—stress-inducible molecules expressed on tumor cells—has enrolled patients with UC. Autologous T cells engineered to express an NKG2D-based CAR demonstrated an acceptable safety profile; however, clinical efficacy in bladder cancer remains to be established (5). A novel German-developed strategy involves engineering the NKG2D receptor into CAR-T cells to improve tumor infiltration, and this approach is currently being evaluated in an active clinical trial (NCT04245824).
- Others (PSMA, and FRα**):** Early-phase clinical trials (NCT03185468) are evaluating CAR-T therapies targeting prostate-specific membrane antigen (PSMA) and folate receptor-α (FRα) in patients with advanced UC. These “fourth-generation” CAR-T constructs incorporate cytokine-secreting capabilities to improve T-cell persistence and antitumor activity. Clinical outcomes from these trials remain pending.

Despite these encouraging preclinical and early clinical advances, the majority of CAR-T strategies targeting UC are still confined to preclinical investigation or early-phase clinical trials, and robust long-term efficacy data are currently unavailable. Compared with hematologic malignancies, CAR-T therapy in solid tumors—including bladder cancer—faces additional challenges: tumor antigen heterogeneity, poor T-cell infiltration into the tumor parenchyma, and the immunosuppressive TME. Treatment-related toxicities, such as cytokine release syndrome and immune effector cell–associated neurotoxicity syndrome, also remain important clinical considerations. Furthermore, the complexity of CAR-T manufacturing and high production costs present logistical barriers to widespread clinical implementation. Ongoing research efforts focusing on next-generation armored CAR constructs, dual-target CAR designs, and combinatorial immunotherapy strategies may help overcome these limitations and improve therapeutic durability.

CAR-NK/CAR-γδ T cells: Natural killer (NK) or γδ T cells can be genetically engineered as “CAR-NK” or “CAR-γδ”. These engineered cells harness the inherent cytotoxic activity of innate immune effectors while exhibiting a reduced risk of graft-versus-host disease. These are experimental in bladder cancer.

#### 3.2.2 Novel cellular therapies

Other adoptive cell therapy strategies include tumor-infiltrating lymphocyte (TIL) therapy and dendritic cell–based vaccines. A study conducted in Tehran reported a HER2-targeted dendritic cell vaccine for metastatic UC, demonstrating favorable safety and preliminary evidence of immune responses (18). These personalized cell therapies remain in early-stage development for UC.

### 3.3 Other emerging immunotherapies

#### 3.3.1 Oncolytic viruses and gene therapy

Oncolytic viruses (OVs) are engineered or naturally occurring agents that selectively infect and kill tumor cells while concurrently stimulating antitumor immune responses. Several OVs are currently undergoing clinical evaluation in bladder cancer:

- CG0070 (CytomX): An adenovirus engineered to replicate in Rb-pathway-deficient cells and secrete granulocyte-macrophage colony-stimulating factor (GM-CSF). A phase II trial in BCG-unresponsive NMIBC (CIS, Ta/T1) showed a 47% complete response (CR) rate overall; notably, the CR rate reached 83.7% among patients with CIS alone, with a favorable safety and tolerability profile (11). CG0070 is currently under further study and has received FDA Fast Track designation.
- Nadofaragene firadenovec (Adstiladrin): An intravesical adenoviral vector that delivers the interferon-α2b gene. Approved by the U.S. FDA in 2022 for BCG-unresponsive CIS, this therapy achieved 51% CR at 3 months and 46% of responders remained in CR at 12 months (9). It represents a pioneering example of immunogene therapy in UC.
- Ornatasvir (ONYX-015): A mutant adenovirus tested years ago in NMIBC, but limited by pre-existing immunity. Next-generation OVs with enhanced tumor-targeted delivery and immune evasion properties are currently under development.

#### 3.3.2 Cytokine and immune modulators

- N-803 (Nogapendekin alfa-inbakicept): An IL-15 superagonist fused to the sushi domain of IL-15Rα. In patients with BCG-unresponsive CIS, intravesical co-administration of N-803 with BCG achieved a CR rate of 62%, substantially exceeding historical CR rates observed with intravesical BCG monotherapy. The median duration of response was significantly prolonged, with 58% of initial responders maintaining CR at 12 months. This approach invigorates NK and T cells and improves BCG efficacy (1).
- Toll-like receptor (TLR) agonists: Compounds such as imiquimod (a TLR7/8 agonist) and poly-ICLC (a TLR3 agonist) are being tested intravesically to mimic viral infection and activate the innate immune response. Phase I clinical trials have demonstrated an acceptable safety profile, however, efficacy data remain pending.
- Bispecific T-cell Engagers (BiTEs): Bispecific antibodies that simultaneously engage T cells—via CD3—and tumor-associated antigens represent an emerging therapeutic modality. In UC, a phase I trial of the PSMA–CD3 bispecific antibodies (REGN5678) demonstrated early evidence of antitumor activity. The clinical success of sacituzumab govitecan has further catalyzed interest in bispecifics targeting TROP-2 or NECTIN-4 along with CD3 (19).

Collectively, these emerging therapeutic modalities exemplify the rapidly expanding immunotherapy landscape in bladder cancer. A structured overview of representative emerging immunotherapeutic targets, investigational agents, and next-generation cellular therapy platforms is presented in **Table 1**.

**Table 1:** Emerging immunotherapy targets and agents in bladder cancer.

Agent Name	Target	Modality	Development Stage	Key Trial Results/Notes
Antibody-drug conjugates (ADCs)				
Enfortumab vedotin (Padcev)	Nectin-4	ADC (MMAE payload)	Approved	EV-302 (Phase 3): In combination with pembrolizumab, significantly improved median OS (31.5 vs. 16.1 months) and PFS compared to chemotherapy in previously untreated locally advanced or metastatic urothelial carcinoma (la/mUC).
Sacituzumab govitecan (Trodelvy)	Trop-2	ADC (SN-38 payload)	Approved	TROPHY-U-01 (Phase 2): Showed an ORR of 27% in heavily pre-treated mUC patients. TROPiCS-04 (Phase 3): Did not meet the primary endpoint of OS benefit versus single-agent chemotherapy in previously treated la/mUC, though numerical improvements in ORR and PFS were observed.
Disitamab vedotin (RC48)	HER2	ADC (MMAE payload)	Late-stage Clinical	RC48-C016 (Phase 3): In combination with toripalimab, demonstrated statistically significant improvement in PFS and OS compared to chemotherapy in HER2-expressing la/mUC. Granted Breakthrough Therapy Designation by FDA.
Novel immunomodulators				
N-803 (Anktiva)	IL-15Rα/IL-2Rβγ	IL-15 superagonist complex	Approved	QUILT-3.032 (Phase 2/3): Approved in combination with BCG for BCG-unresponsive non-muscle invasive bladder cancer (NMIBC) with carcinoma *in situ* (CIS). Showed a complete response rate of 71% with durable responses.
Bispecific antibodies & T-Cell engagers				
RNDO-564	Nectin-4 x CD28	Bispecific Antibody (co-stimulatory)	Preclinical/Phase 1 planned	Preclinical data shows potent T-cell activation and anti-tumor activity. Designed to provide a co-stimulatory signal specifically at the tumor site. A Phase 1 first-in-human study is planned.
CLSP-1025	p53 R175H x CD3	T-cell engaging bispecific	Phase 1	Currently in a Phase 1 dose-escalation study for patients with advanced solid tumors, including bladder cancer, harboring the specific p53 R175H mutation.
Cell therapies & vaccines				
RUTI Vaccine	Broad immune stimulation	Therapeutic Vaccine (non-live TB bacteria)	Early Phase Clinical	RUTIVAC-1 (Pilot Study): Showed potential to enhance the immune response to BCG and improve recurrence-free survival in patients with high-risk NMIBC.
4SCAR-T Cells	PSMA, FRα, others	CAR T-cell Therapy	Early Phase Clinical	NCT03185468 (Phase 1/2): An ongoing trial evaluating fourth-generation CAR-T cells targeting antigens like PSMA in patients with advanced or metastatic urothelial bladder cancer. Clinical results are not yet available.

ADC, Antibody-Drug Conjugate; BCG, Bacillus Calmette-Guérin; CAR, Chimeric Antigen Receptor; CIS, Carcinoma *In Situ*; FRα, Folate Receptor alpha; HER2, Human Epidermal Growth Factor Receptor 2; IL-15, Interleukin-15; MMAE/SN-38, Cytotoxic drug payloads; NMIBC, Non-Muscle Invasive Bladder Cancer; ORR, Objective Response Rate; OS, Overall Survival; PFS, Progression-Free Survival; PSMA, Prostate-Specific Membrane Antigen.

### 3.4 Combination therapeutic strategies

Given that monotherapy responses are often partial, combinatorial regimens are an active focus. These combinatorial strategies aim to convert “cold” tumors into “hot” tumors, overcome immunosuppressive pathways, and achieve more durable clinical remissions.

- ICI + chemotherapy: Combining PD-1/PD-L1 inhibitors with platinum-based chemotherapy was logical, but phase III trials (IMvigor130, KEYNOTE-361) failed to show significant OS benefit over chemotherapy alone (3). However, the CheckMate-901 trial (gemcitabine/cisplatin ± nivolumab) reportedly met PFS and OS primary endpoints in 2023, suggesting potential synergistic activity in cisplatin-eligible patients (3). Ongoing studies will clarify if chemotherapy can prime immunogenic cell death enhancing ICI effect.
- ICI + ADC: The success of EV in combination with pembrolizumab represents a landmark achievement in oncology (5, 6). Similarly, sacituzumab govitecan (an anti-Trop-2 ADC) is currently being evaluated in combination with pembrolizumab in the TROPHY-U-01 trial. The rationale is that ADCs kill tumor cells and release neoantigens, while the antibody component may engage Fc receptors to activate immunity. Early clinical data from the EV-103 trial demonstrate deep and durable responses with the ADC–ICI combination (6).
- Dual checkpoint blockade: Combining PD-1/PD-L1 inhibitors with CTLA-4 inhibitors may enhance antitumor efficacy, albeit with the expense of increased immune-related toxicity. The DANUBE trial (durvalumab + tremelimumab) did not achieve co-primary endpoints, although a trend in PD-L1–positive tumors was noted (3). The ongoing CheckMate-901 trial (nivolumab + ipilimumab) has yielded mixed interim results. In NMIBC, trials of ICIs with intravesical BCG (e.g. pembrolizumab + BCG) or therapeutic vaccines are exploring additive effects.
- ICI + radiation: Radiotherapy can increase tumor antigen release. A small phase II trial evaluating the combination of stereotactic radiotherapy with dual ICIs (durvalumab + tremelimumab) in patients with MIBC reported a remarkable 81% complete response rate, thereby enabling bladder preservation (20).These findings suggest robust synergistic activity, however, validation in larger and confirmatory studies is warranted.
- ICI + targeted therapy: Combining immunotherapy with agents targeting oncogenic drivers is promising. Notably, in the FORT-2 trial, the combination of rogaratinib (an FGFR inhibitor) and atezolizumab achieved an ORR of 54% in patients with FGFR-positive, cisplatin-ineligible metastatic UC—a marked improvement over the ORRs observed with either agent as monotherapy (23% with atezolizumab alone and 21% with rogaratinib alone) (21). These findings suggest a potential synergistic interaction between FGFR inhibition and PD-L1 blockade in this biomarker-selected population.
- ADC + other agents: Disitamab vedotin (RC48) in combination with anti–PD-1 antibody showed higher response than either alone (22); similarly, the combination of sacituzumab govitecan and pembrolizumab showed enhanced efficacy (TROPHY trial) (23). ADCs directed against novel tumor-associated antigens are currently in trials—for example, FGFR3-targeted ADCs and PSMA- targeted ADCs.
- Intravesical combination Therapy: In NMIBC, several phase III trials are currently evaluating the efficacy of intravesical BCG combined with systemic ICIs—such as sasanlimab or pembrolizumab—in patients with BCG-naïve, high-risk disease. Preliminary data indicate that these combination regimens are feasible; notably, the sasanlimab + BCG regimen recently met its primary endpoint of event-free survival (24). Another promising approach is the combination of intravesical IL-15 agonist N-803 with BCG (25–27).
- Novel bispecifics and fusion proteins: Bispecific inhibitors targeting PD-1/CTLA-4 or TGF-β/PD-L1—such as bintrafusp alfa—have been evaluated in clinical studies. Bintrafusp alfa (not bladder-specific), a bifunctional fusion protein that simultaneously blocks PD-L1 and TGF-β, demonstrated only modest antitumor activity across multiple non-bladder malignancies (28).

## 4 Mechanisms of resistance to immunotherapy

### 4.1 Tumor microenvironment and immunosuppression

The TME in UC plays a critical role in modulating immune evasion, therapeutic resistance, and clinical outcomes. Multiple immunosuppressive cellular and molecular components collectively drive tumor progression and influence immunotherapy responsiveness.

### 4.2 Immune cellular resistance

Tumor-associated macrophages (TAMs) represent one of the predominant immune regulatory cell populations within the bladder cancer TME. TAMs commonly exhibit an M2-polarized phenotype, characterized by the secretion of vascular endothelial growth factor (VEGF), interleukin-10 (IL-10), transforming growth factor-β (TGF-β), and arginase-1, which collectively suppress cytotoxic T-cell activity and promote tumor progression (29, 30). Recent clinical and translational studies have demonstrated that elevated infiltration of M2-like macrophages is significantly associated with advanced tumor stage, impaired T-cell activation, and poorer survival outcomes in patients with UC (31). Furthermore, specific TAM subsets expressing markers such as CD163 and MS4A4A have been implicated in remodeling the TME toward an immunosuppressive state and in promoting T-cell exhaustion, thereby reinforcing their critical role in mediating resistance to immunotherapy (32).

Regulatory T cells (Tregs) further contribute to immune suppression through the expression of inhibitory immune checkpoint molecules—such as CTLA-4—and the secretion of anti-inflammatory cytokines including IL-10 and TGF-β (33). Several cohort-based studies have demonstrated that elevated intratumoral Treg infiltration in bladder cancer is associated with increased recurrence risk and reduced therapeutic responsiveness, including resistance to BCG and ICIs (34, 35).

### 4.3 Stromal-mediated immune exclusion

Stromal-mediated immune exclusion represents a major barrier to effective antitumor immunity. Cancer-associated fibroblasts (CAFs) drive extracellular matrix remodeling and secrete TGF-β, thereby imposing physical constraints on immune cell infiltration and inducing T-cell dysfunction (36, 37). Elevated stromal TGF-β signaling is strongly associated with immune-excluded tumor phenotypes and diminished response to ICI across multiple solid malignancies, including bladder cancer (38). Moreover, CAF-derived gene expression signatures correlate significantly with disease progression and reduced survival, highlighting the translational importance of stromal immunoregulation in UC (39).

### 4.4 Metabolic resistance pathways

Beyond cellular and stromal factors, metabolic reprogramming within the TME plays a critical role in driving immune dysfunction. Hypoxia, lactate accumulation, and adenosine-mediated signaling collectively suppress effector T-cell proliferation and cytokine production while promoting regulatory immune cell phenotypes (40–42). Emerging translational evidence further indicates that these metabolic alterations contribute to resistance to ICIs and promote disease progression in bladder cancer (43, 44).

Collectively, the dynamic interplay among immunosuppressive immune cells, stromal exclusion mechanisms, and metabolic dysregulation highlights the multifaceted nature of immune resistance in UC. Targeting these TME components is increasingly recognized as a promising strategy to enhance the efficacy of immunotherapy and improve clinical outcomes. A summary of major immune resistance mechanisms and their corresponding therapeutic strategies is presented in **Table 2**.

**Table 2:** Major immune resistance mechanisms and corresponding therapeutic strategies in bladder cancer.

Resistance mechanism category	Key cellular/Molecular drivers	Mechanism of immune evasion	Emerging therapeutic strategies	Representative investigational approaches
Immune cellular resistance	TAMs (M2 phenotype)	Suppression of cytotoxic T-cell activation; promotion of tumor angiogenesis	TAM reprogramming; macrophage depletion; CSF-1R inhibition	CSF-1R inhibitors; CD47–SIRPα blockade
	Regulatory T cells (Tregs)	CTLA-4-mediated T-cell suppression; anti-inflammatory cytokine release	Treg depletion; checkpoint inhibitor combinations	CTLA-4 inhibitors; combination ICIs
	Myeloid-derived suppressor cells (MDSCs)	Inhibition of T-cell proliferation; cytokine-mediated immune suppression	MDSC-targeting agents; immune checkpoint combinations	CXCR2 inhibitors; STAT3 pathway inhibitors
Stromal-mediated immune exclusion	Cancer-associated fibroblasts (CAFs)	Physical stromal barriers; extracellular matrix remodeling	CAF-targeting therapies; stromal normalization	FAP-targeted therapies; anti-fibrotic agents
	TGF-β signaling	T-cell exclusion from tumor nests; immune suppression	TGF-β inhibitors; TGF-β/ICI combination therapies	Bintrafusp alfa; TGF-β pathway inhibitors
Metabolic resistance pathways	Hypoxia-driven signaling	Reduced antigen presentation; induction of immune checkpoint expression	Hypoxia-targeted therapies; angiogenesis normalization	HIF pathway inhibitors; anti-angiogenic combinations
	Lactate accumulation	Promotion of immunosuppressive macrophage polarization; T-cell inhibition	Metabolic reprogramming strategies	LDH inhibitors; metabolic checkpoint targeting
	Adenosine signaling	A2A receptor-mediated T-cell suppression; expansion of regulatory immune cells	Adenosine pathway blockade	A2A receptor antagonists; CD73 inhibitors
Tumor intrinsic resistance	Antigen presentation defects	Reduced tumor antigen recognition by T cells	Tumor vaccine strategies; neoantigen targeting	Personalized cancer vaccines; mRNA vaccines
	Alternative immune checkpoints (LAG-3, TIGIT, TIM-3)	T-cell exhaustion and immune escape	Next-generation checkpoint blockade	LAG-3 inhibitors; TIGIT inhibitors

Major immune resistance mechanisms contributing to immunotherapy failure in bladder cancer and corresponding emerging therapeutic strategies. TAMs, tumor-associated macrophages; Tregs, regulatory T cells; MDSCs, myeloid-derived suppressor cells; CAFs, cancer-associated fibroblasts; TGF-β, transforming growth factor-beta; HIF, hypoxia-inducible factor.

## 5 Biomarkers of treatment response

Given the heterogeneity in treatment responses, identifying predictive biomarkers is essential. Recent studies have highlighted:

- Genetic signatures: A meta-analysis by Boll et al. integrated clinical and molecular data from 707 patients with advanced bladder cancer who received ICIs. The analysis confirmed that tumor mutational burden (TMB) exhibits a positive yet modest correlation with treatment response, whereas the association between PD-L1/PD-1 expression and response remained inconsistent across studies. Notably, the neuronal molecular subtype was associated with significantly higher response rates, highlighting the potential clinical utility of subtype-informed therapeutic strategies (7).
- Tumor microenvironment signatures: Gene expression profiles of immune cell markers—including CD8A, PD-L1, and IFN-γ–related genes—can collectively define “hot tumor” signatures. Váradi et al. identified an “immune cell signature” predictive of therapeutic response. Elements such as CXCL12 and C3, identified in that study, suggest chemokine milieu is relevant (45). The NeoPeptide trial (NCT03012344) is evaluating signatures from on-treatment biopsies to refine predictors.
- Circulating tumor DNA (ctDNA): ctDNA is a promising biomarker in bladder cancer. Multiple studies have shown the clinical potential of ctDNA in bladder cancer for early diagnosis, prognosis assessment, early detection of minimal residual disease (MRD), disease recurrence, and treatment-response monitoring (46). In the adjuvant immunotherapy setting, ctDNA enables better prognostic stratification and better patient selection for adjuvant treatment. ctDNA dynamics can be employed to evaluate treatment responses. A decrease or clearance of ctDNA might indicate treatment responses, while an increase or no change in ctDNA levels might suggest treatment resistance (46–50). Longitudinal monitoring of ctDNA during treatment can enable timely and precise adjustments to the treatment strategy, thereby facilitating personalized treatment guidance (46).
- Combined biomarker models: Multi-parameter predictive models—incorporating TMB, PD-L1 expression, gene expression profiles, and clinical variables—are under active development. For instance, multiple studies found that microsatellite instability (MSI) is associated with better response to ICIs across a variety of solid malignancies (2, 51). However, Bonneville et al. reported MSI-H was detected in only 0.49% of bladder cancer cases (52). Thus, the generalizability of this finding to bladder cancer warrants further investigation. DNA damage response and repair (DDR) defects play an important role in the occurrence, development, and treatment-response to immunotherapy of bladder cancer. Teo et al. reported that DDR alterations were associated with a higher immunotherapy response rate (67.9% vs. 18.8%, *P* < 0.001) as well as improved OS and PFS over DDR mutations. Notably, patients harboring deleterious DDR mutations exhibited a higher response rate than those with DDR alterations (80% vs. 54%, *P* < 0.001) (53). However, conclusive evidence from randomized trials is still lacking to support the role of DDR genes in predicting ICI response for patients with bladder cancer.

Overall, in bladder cancer immunotherapy, biomarkers can be categorized by clinical validation. PD-L1 expression is the most widely used clinically validated biomarker incorporated into regulatory-approved decisions, though its predictive accuracy is imperfect (2). TMB is also regulatory-approved in certain settings, reflecting tumor immunogenicity, but its predictive value varies across studies (2, 7). Beyond established biomarkers, numerous emerging ones are under active investigation to refine patient selection for personalized immunotherapy. ctDNA show promise for dynamic treatment assessment. Immune cell signatures, spatial TME profiling, and microbiome-related biomarkers also demonstrate translational potential. However, these remain investigational and require prospective validation. Ultimately, integrating multi-omic biomarkers with TME characterization and dynamic monitoring may provide more personalized immunotherapy guidance in bladder cancer.

## 6 Critical evaluation of key clinical trials and therapeutic strategies

A multitude of clinical trials has shaped current clinical practice and continues to guide future research. Here we review some highlights:

- IMvigor210/211 (atezolizumab): The Phase II IMvigor210 trial showed 15% ORR with atezolizumab in second-line UC, independent of PD-L1 expression status. In contrast, the Phase III IMvigor211 trial compared atezolizumab versus chemotherapy in patients with platinum-refractory UC and failed to meet its primary endpoint (OS) in the PD-L1–positive subgroup (54). Although median OS was numerically longer with atezolizumab than with chemotherapy (11.1 vs. 10.6 months), this difference did not reach statistical significance. Consequently, atezolizumab monotherapy remains a treatment option following platinum-based chemotherapy, albeit without confirmed OS benefit in a pivotal Phase III setting. Furthermore, the Phase III IMvigor130 trial—which evaluated atezolizumab plus chemotherapy versus chemotherapy alone—showed improvement in PFS but no significant OS benefit, thereby limiting the impact of this combination regimen (5, 15).
- KEYNOTE Trials (pembrolizumab): KEYNOTE-052 (Phase II), a study in cisplatin-ineligible patients with first-line UC, demonstrated an ORR of approximately 29% with pembrolizumab monotherapy. KEYNOTE-361 (Phase III) evaluated pembrolizumab alone or in combination with chemotherapy versus chemotherapy alone in first-line UC; however, neither pembrolizumab-containing arm achieved a statistically significant improvement in overall survival (3). KEYNOTE-045 (Phase III), conducted in second-line UC, showed a statistically significant OS benefit for pembrolizumab versus chemotherapy (10.3 vs. 7.4 months), establishing pembrolizumab as second-line standard (5). Additionally, KEYNOTE-052 data supported the use of pembrolizumab in high-risk NMIBC following BCG therapy, with a CR rate of 41% (8).
- CheckMate Trials (nivolumab): CheckMate-275 (Phase II) demonstrated an ORR of 19.6% in patients with metastatic UC following platinum-based chemotherapy. CheckMate-274 (Phase III) showed that adjuvant nivolumab significantly improved DFS compared with placebo (21.0 vs. 10.9 months) in patients with high-risk MIBC after radical cystectomy (12, 13). CheckMate-901 (nivolumab in combination with first-line chemotherapy) recently met both PFS and OS primary endpoints, potentially establishing a new ICI-based combination option for fit patients.
- DANUBE (durvalumab ± tremelimumab): This large, randomized, Phase III trial evaluated first-line treatment in cisplatin-eligible patients with UC, comparing durvalumab monotherapy, durvalumab + tremelimumab, and chemotherapy. The trial did not meet its co-primary endpoints—OS in the PD-L1–positive subgroup and OS in all-comers. Nevertheless, median OS was numerically longer in the dual-blockade arm (15.1 months) than in the durvalumab monotherapy arm (14.4 months), suggesting a potential clinical signal (3).
- JAVELIN Bladder 100 (avelumab maintenance): This trial randomized patients without progression after first-line chemotherapy to receive either avelumab maintenance therapy or best supportive care. Median OS was significantly improved with avelumab versus control (21.4 vs. 14.3 months, HR 0.69) (55).Updated data further confirm prolonged benefit, with median OS extending to approximately 24 months with avelumab maintenance, and the safety profile remains favorable and manageable (4). Avelumab maintenance therapy is now established as the standard of care in this setting.
- EV-301 (Enfortumab Vedotin, EV): For patients with prior platinum-based chemotherapy and PD-1/PD-L1 inhibitors, the Phase III EV-301 trial showed that EV significantly improved OS compared with investigator’s choice of chemotherapy (median OS 12.9 vs. 9.0 months; HR 0.70). In the confirmatory trial, the ORR was 44% with EV versus 12% with chemotherapy; the most common adverse events were manageable neuropathy and rash (5).
- EV-302/KEYNOTE-A39: A practice-changing, first-line trial in cisplatin-ineligible UC. EV plus pembrolizumab demonstrated unprecedented efficacy, with a median OS of 31.5 months versus 16.1 months with chemotherapy (HR 0.47) (56). This combination has established a new standard of care; the U.S. FDA granted full approval in 2023. Notably, in the lead-in phase of the EV-103 trial (Cohort K), EV plus pembrolizumab achieved an ORR of 67.8%, with durable responses (median duration of response: 22.1 months), compared with an ORR of 45.2% for EV monotherapy (6).
- TROPHY-U-01 (Sacituzumab Govitecan, SG): This phase II study in patients with metastatic UC reported an ORR of 27% and a median OS of 10.9 months (57). The subsequent phase III trial, TROPiCS-04, compared SG with the investigator’s choice of chemotherapy in patients with advanced UC who had progressed after platinum-based chemotherapy and PD-1/PD-L1 inhibitors. Unfortunately, SG did not meet its co-primary endpoint of OS and PFS (58).
- Neoadjuvant Trials: Several trials evaluating ICIs in combination with cisplatin-based chemotherapy for MIBC—including BLASST-1 and NABUCCO—have demonstrated promising pathological complete response rates (up to 50%), however, the long-term benefit remains variable across studies (59). CheckMate-274 demonstrated a benefit in the adjuvant setting, suggesting that neoadjuvant ICI (as being evaluated in ongoing trials such as MK-3475 and MK-2775) may improve cure rates in resectable disease, pending the reporting of full trial results (12, 60).

Pivotal clinical trials evaluating ICIs and combination therapeutic strategies in bladder cancer are summarized in **Table 3**. Overall, ICIs have yielded durable remissions in a subset of patients, however, not all individuals respond. Long-term survivorship analyses indicate that some patients achieve sustained disease control for multiple years (7, 14). Nevertheless, a substantial proportion of tumors remain refractory to ICI therapy. Understanding the biological basis of treatment response—such as the distinction between immunologically “hot” and “cold” tumors (7, 45)—and refining patient selection criteria remain critical.

**Table 3:** Key pivotal clinical trials defining bladder cancer immunotherapy.

Trial name	Phase	Setting	Intervention	Main outcome(s)/Key findings
Metastatic setting (frontline & maintenance)				
EV-302/KEYNOTE-A39	3	1L la/mUC (cisplatin-eligible and ineligible)	Enfortumab vedotin + Pembrolizumab *vs.* Chemotherapy (Gemcitabine + Cisplatin/Carboplatin)	New 1L Standard of Care: Demonstrated superior Overall Survival (median OS 31.5 vs. 16.1 mo) and Progression-Free Survival (median PFS 12.5 vs. 6.3 mo) compared to standard chemotherapy, regardless of cisplatin eligibility or PD-L1 status.
JAVELIN Bladder 100	3	1L Maintenance mUC (after non-progression on platinum chemo)	Avelumab + Best Supportive Care (BSC) *vs.* BSC alone	Maintenance Standard: Significantly prolonged Overall Survival (median OS 21.4 vs. 14.3 mo) in patients whose disease had not progressed after 4–6 cycles of 1L platinum-based chemotherapy.
IMvigor130	3	1L mUC (cisplatin-eligible and ineligible)	Atezolizumab + Chemotherapy *vs.* Atezolizumab mono *vs.* Chemotherapy + Placebo	Complex Results: Met co-primary endpoint of improved PFS for Atezo+Chemo vs. Chemo alone. However, did not show a statistically significant improvement in Final OS for the combination arm versus chemotherapy alone.
Metastatic setting (second-line+)				
KEYNOTE-045	3	2L mUC (post-platinum failure)	Pembrolizumab *vs.* Investigator’s Choice Chemotherapy	Established 2L Standard: Demonstrated superior Overall Survival (median OS 10.3 vs. 7.4 mo) compared to chemotherapy in patients who progressed after platinum-based therapy.
IMvigor211	3	2L mUC (post-platinum failure)	Atezolizumab *vs.* Investigator’s Choice Chemotherapy	Did Not Meet Primary Endpoint: Failed to show a statistically significant OS benefit in the primary analysis population (high PD-L1 expression), though clinical activity was observed in the ITT population.
Muscle-invasive setting (adjuvant)				
CheckMate-274	3	Adjuvant MIBC (high-risk post-radical surgery)	Nivolumab *vs.* Placebo	Adjuvant Standard: Significantly improved Disease-Free Survival (DFS) in the Intent-to-Treat (ITT) population (median DFS 20.8 vs 10.8 mo) and patients with PD-L1 ≥1%.
AMBASSADOR (A031501)	3	Adjuvant MIBC (high-risk post-radical surgery)	Pembrolizumab *vs.* Observation	Positive DFS: Met primary endpoint showing a statistically significant improvement in Disease-Free Survival compared to observation in high-risk patients after surgery.
Non-muscle invasive setting (NMIBC)				
KEYNOTE-057 (Cohort A)	2	BCG-unresponsive NMIBC with CIS (with or without papillary tumors)	Pembrolizumab (monotherapy)	FDA Approval Basis: Single-arm study showing a Complete Response (CR) rate of approximately 41% at 3 months, with a median duration of response of 16.2 months.
QUILT-3.032	2/3	BCG-unresponsive NMIBC with CIS (with or without papillary tumors)	N-803 (Anktiva) + BCG	FDA Approval Basis: Single-arm study showing a high Complete Response (CR) rate of 71%, with durable responses observed at 12 and 24 months.

1L/2L, First-line/Second-line; BCG, Bacillus Calmette-Guérin; BSC, Best Supportive Care; CIS, Carcinoma *In Situ*; CR, Complete Response; DFS, Disease-Free Survival; ITT, Intent-to-Treat; la/mUC, Locally Advanced or Metastatic Urothelial Carcinoma; MIBC, Muscle-Invasive Bladder Cancer; NMIBC, Non-Muscle Invasive Bladder Cancer; OS, Overall Survival; PFS, Progression-Free Survival.

## 7 Future directions and perspectives

Immunotherapy has significantly transformed the therapeutic landscape for bladder cancer, however, durable responses remain limited to a subset of patients. This marked heterogeneity highlights the urgent need to refine current treatment strategies and explore novel approaches. Moving forward, future research efforts are increasingly focused on optimizing the sequencing and combination of immunotherapeutic regimens and advancing next-generation immune-engineered cellular therapies.

### 7.1 Optimizing therapeutic sequencing and combination strategies

Retreatment and rechallenge strategies following ICI resistance have emerged as an area of active investigation. Early-phase clinical data suggest that selected patients may derive benefit from ICI retreatment after treatment interruption or subsequent systemic therapy, potentially through reactivation of antitumor immunity or restoration of immune sensitivity (61, 62). However, validated predictive biomarkers and evidence-based patient selection criteria remain lacking, highlighting the critical need for prospective trials with comprehensive correlative analyses (63, 64). Concurrently, combination strategies are being rigorously pursued to overcome both primary and acquired resistance. Certain ADC–ICI combination regimens have demonstrated particularly promising efficacy (56, 65). ICIs are also under active evaluation in combination with targeted therapies, radiotherapy, and intravesical immunomodulators, with emerging evidence supporting the potential of multimodal immune activation to extend therapeutic benefit (66, 67). Mechanistic insights—including the identification of TGF-β–mediated immune-excluded phenotypes—further inform the rational design of combination regimens aimed at remodeling the immunosuppressive TME and restoring immune responsiveness (38).

### 7.2 Next-generation cellular immunotherapies

Next-generation Cellular immunotherapy represents a rapidly evolving frontier. CAR-T cells targeting tumor-associated antigens including NECTIN4, PSMA, and HER2 are under early-stage clinical evaluation, with preclinical and early-phase clinical evidence supporting potent and tumor-specific cytotoxicity (17, 68, 69). Additionally, emerging chimeric antigen receptor–natural killer (CAR-NK) and “armored” CAR-T cell technologies—incorporating cytokine-secreting capabilities or checkpoint resistance—aim to enhance tumor infiltration and overcome immunosuppressive TME (70).

### 7.3 Personalized vaccines and novel targets

Personalized cancer vaccines are emerging as a promising immunotherapeutic strategy. Recent translational studies evaluating neoantigen-based vaccines combined with ICIs have demonstrated the capacity to induce durable, antigen-specific T-cell responses in UC (71, 72). Meanwhile, novel immunomodulatory targets within the TME—including LAG-3, TIGIT, TIM-3, and stromal or metabolic immunosuppressive pathways—are under active investigation, with early studies suggesting that targeting these pathways may enhance the efficacy of ICIs (73, 74).

**Figure 3:**
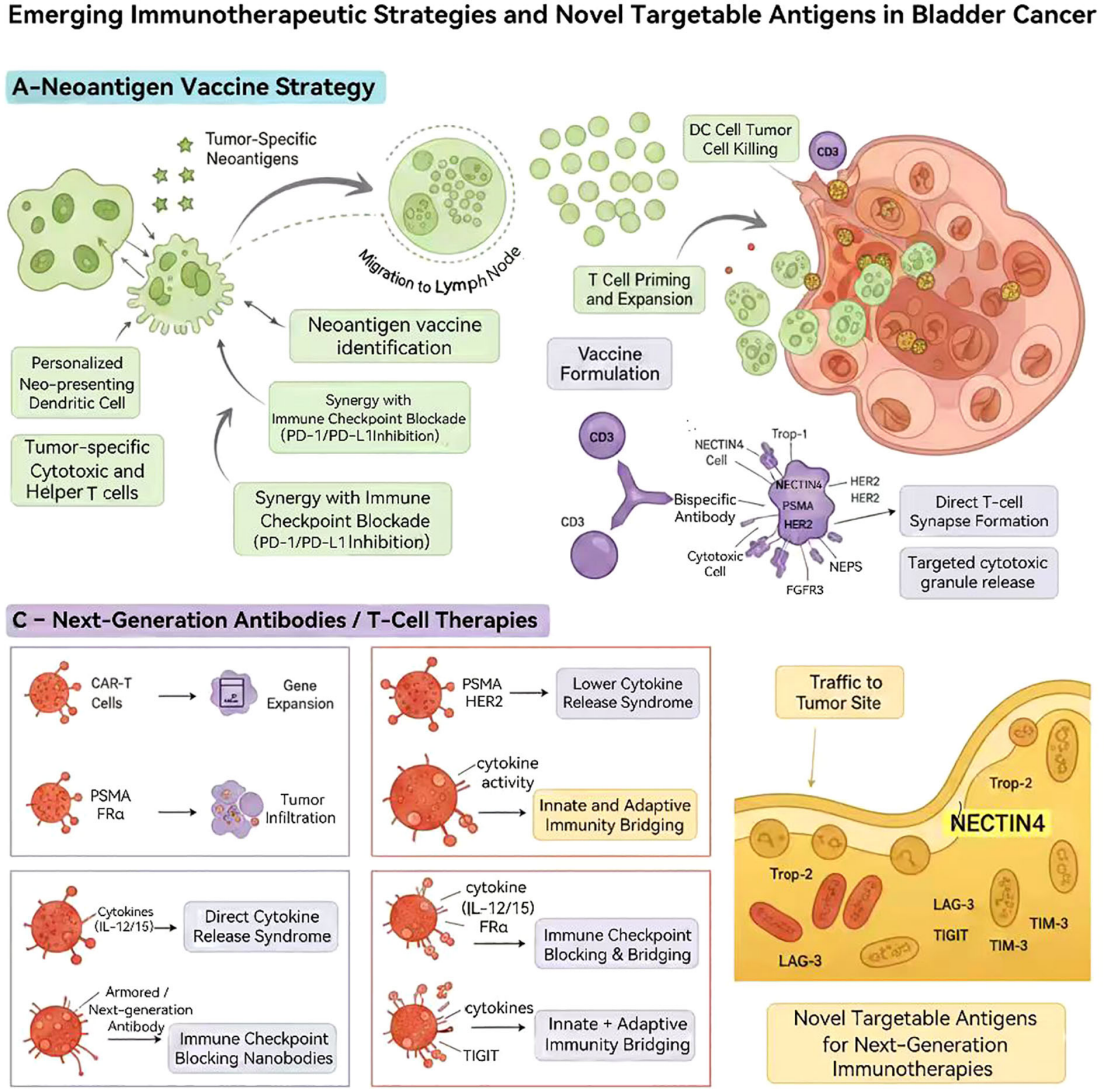
Emerging immunotherapeutic strategies and novel targetable antigens in bladder cancer.

To offer a comprehensive conceptual synthesis of these emerging immunotherapeutic approaches, **Figure 3** provides a schematic overview of next-generation strategies and novel targetable antigens in bladder cancer. This integrated framework elucidates how personalized vaccines, bispecific antibodies, and engineered cellular therapies can act synergistically to enhance tumor-specific immune activation and overcome microenvironmental immune resistance.

In conclusion, immunotherapy has brought unprecedented advances in the treatment of bladder cancer, significantly improving survival outcomes for many patients (4, 6). Ongoing advances in combination regimens, biomarker-guided precision approaches, and next-generation immune engineering technologies holds strong promise for further enhancing therapeutic efficacy. Sustained efforts to overcome immune resistance and optimize patient-specific treatment selection will be critical to translate these innovations into durable clinical benefit. Collectively, the rapidly evolving landscape of bladder cancer immunotherapy offers renewed optimism for improvements in patient outcomes.
